# Evaluation of Two Supplemented Culture Media for Long-Term, Room-Temperature Preservation of* Streptococcus pneumoniae* Strains

**DOI:** 10.1155/2017/1218798

**Published:** 2017-11-22

**Authors:** Beatriz Quintero Moreno, María Araque, Evelyn Mendoza

**Affiliations:** ^1^Departamento de Microbiología y Parasitología Clínicas, Facultad de Medicina, Universidad de Los Andes, Mérida 5101, Venezuela; ^2^Laboratorio de Microbiología Molecular, Facultad de Farmacia y Bioanálisis, Universidad de Los Andes, Mérida 5101, Venezuela

## Abstract

**Objective:**

To produce two supplemented agar types in order to store pneumococci for several months at room temperature.

**Methods:**

Todd-Hewitt/Hemoglobin/Yeast/Charcoal/Agar (TH-HYC) and Todd-Hewitt/Skim-Milk/Yeast/Charcoal/Agar (TH-SYC) were used to prepare two supplemented agar types. Nineteen pneumococci isolated from patients or asymptomatic carriers displaying diverse serotypes and multilocus sequence types (MLST) were subcultured and stored onto supplemented agar types, in four different tests, at room temperature.

**Findings:**

At the end of all tests (4–6 months) all noncontaminated subcultures were viable and maintained all phenotypic characteristics. Survival-time curves revealed a slow decrease of viable CFU over time on agar types, but at the end the number of viable CFU was satisfactory (≥2+ of growth). Decreasing of CFU was significantly higher for clinical versus nasopharyngeal isolates. Subcultures contamination rates were 6.25% and 14.58% after 2 and 6 months of storage, respectively.

**Conclusion:**

TH-HYC and TH-SYC agar types allowed the viability of pneumococci with several serotypes, MLST, and genetic profiles, after 6 months of storage at room temperature. We consider that these agar types are a valid alternative to preserve pneumococci over an extended period, especially when methods as cryopreservation or lyophilization are not available, and are useful for transporting strains between laboratories.

## 1. Introduction

*Streptococcus pneumoniae*, the pneumococcus, is thought to be responsible for at least half of all community-acquired pneumonia and otitis media and remains a significant cause of bacteremia and meningitis [1]. It is well recognized that despite advances in medical care and appropriate antibiotic treatments, there is a significant mortality from pneumococcal infections. At present, management of these infections has been potentially complicated by the increasing resistance of isolates to commonly used antibiotics [2]. Moreover, pneumococcal serotypes, clones and patterns of antimicrobial resistance, vary among different regions and countries. Also, in the postvaccine era, the frequencies of serotypes and some genes among pneumococcal population seem to have changed; thus a continued pneumococcal vigilance around the globe is needed [2, 3].

An essential aspect of pneumococcal monitoring is the ability to preserve strains during transportation and also its long-term storage [4]. This is because* S. pneumoniae *can only survive for 3-4 days on blood agar plates and/or chocolate agar plates and do not survive for extended periods in broth; hence there is a need for effective and practical short-term and especially long-term storage methods [5].

Short-term storage methods are appropriate for bacterial isolates that only need to be stored for several days or a few weeks. For instance,* S. pneumoniae* can be stored at room temperature on chocolate agar slants for up to one week and on Dorset Transport medium for approximately six weeks. Pneumococci can also be stored on swabs into silica gel packets for approximately two weeks at 4°C and perhaps slightly shorter at room temperature (25°C) [5]. While long-term storage methods as freezing at −70°C and lyophilization are recognized to be effective techniques, the high cost and difficulty in acquiring and maintaining equipment and test materials prevent their use in low-income regions of the world [4].

*S. pneumoniae* is a highly diverse species with important epidemiological and biological differences [6]. While some pneumococcal serotypes and clones have a tendency to colonize the nasopharynx more often asymptomatically, others are found to be associated with invasive and mucosal disease [6–8]. Some studies suggest that epidemiological or biological characteristics, such as carriage prevalence and invasive disease potential, are linked to bacterial growth [7, 9].

In Venezuela, previous studies on pneumococcal colonization have shown an increase of antimicrobial resistance over the years, with the presence of some multidrug-resistant international clones of* S. pneumoniae,* carrying several transposons [10, 11]. However, the need for monitoring pneumococcal changes must overcome several obstacles, especially those related to the storage of the strains. Therefore, in this work we mix several commercial products in order to produce two supplemented agar types with the aim of storing pneumococci for several months at room temperature. It was also our proposed that these media could maintain the viability of pneumococci isolated from patients or asymptomatic carriers, as well as those related to diverse serotypes and multilocus sequence types (MLST).

## 2. Materials and Methods

### 2.1. Bacterial Strains

We used a total of 19 pneumococcal strains from children aged under 5 years, identified and characterized following phenotypic standard procedures as well as conventional PCR and multilocus sequence typing procedures [5, 10–12]. These strains were classified in two groups. The first group included 4 pneumococci strains: 2 from children with meningitis and conjunctivitis and 2 nasopharyngeal isolates from healthy carriers. The second group consisted of 15 nasopharyngeal strains belonging to a larger collection of 125 pneumococci isolated in 2007 in the city of Mérida, Venezuela, and analyzed in a previous study [11]. These 15 strains, displaying representative characteristics of the pneumococci circulating in this geographical region, included the serotypes/serogroups frequently observed in the collection, as well as the most common MLST types identified. Serotypes, MLST types, resistance patterns, and genetic characteristics of pneumococcal strains used in this study are described in Table 1.* Streptococcus pneumoniae *ATCC49619 strain was used as a control for all experiments. All strains were stored in vials with STGG medium (skim milk, glycerol, glucose, and tryptone soya broth) and frozen at −20°C until subsequent use.

### 2.2. Culture Media Composition and Preparation

Culture media for long-time storage were prepared mixing Todd-Hewitt Broth with several commercial products, in order to produce two new supplemented agar types: Todd-Hewitt/Hemoglobin/Yeast/Charcoal (TH-HYC) and Todd-Hewitt/Skim-Milk/Yeast/Charcoal (TH-SYC).

Supplemented TH-HYC agar consisted of 36.4 gr/Lt Todd-Hewitt Broth (Oxoid), 10 gr/Lt hemoglobin powder (Hi Media), 5 gr/Lt yeast extract powder (Oxoid), 4 gr/Lt activated charcoal, and 15 gr/Lt bacteriological agar (Oxoid), in distilled water. Supplemented TH-SYC agar consisted of 36.4 gr/Lt Todd-Hewitt Broth (Oxoid), 100 gr/Lt skim milk powder (Oxoid), 5 gr/Lt yeast extract powder (Oxoid), 4 gr/Lt activated charcoal, and 15 gr/Lt bacteriological agar (Oxoid), in distilled water.

In order to prepare TH-HYC agar, an initial mixture of Todd-Hewitt Broth, yeast extract, and bacteriological agar was dissolved in distilled water by shaking and heating until boiling. Activated charcoal and hemoglobin were dissolved separately in distilled water at room temperature and included into the initial mixture by shaking until the mixture was completely homogenized. The same procedure was followed to prepare TH-SYC agar, except for the addition of skim milk powder instead of hemoglobin. Media were sterilized by autoclaving at 121°C for 15 min and aseptically dispensed into Petri dishes (15 × 100 mm), screw-cap glass tubes (20 × 150 mm), and safe-lock microtubes (1.5-mL). Glass tubes were tilted to create a slant-agar. While pouring the media, bottles were swirled to avoid charcoal settling. Sterility testing was performed after each instance of medium preparation. The sterilized media were stored (6 to 8°C) until further use.

### 2.3. Inoculation Procedure

Prior to testing, one vial of each strain was removed from the freezer and subcultured twice on Brain Heart Infusion (BHI) blood agar plates (Oxoid) and incubated for 24 h at 37°C in a microaerophilic atmosphere, in order to ensure its purity and vitality. The inoculation procedure was carried out according to the recipient the media were poured in. The inoculation procedure for pneumococci kept into plates or glass tubes was as follows: a portion of the blood culture was taken with a 0.001 mL calibrated loop and streaked out onto the surface of the plates or glass tubes with supplemented media. A 4-quadrant streaking method was adopted to inoculate the plates. The tubes, partially closed, and the plates were incubated for 24 h at 37°C in a microaerophilic atmosphere. In order to inoculate pneumococci into microtubes, a portion of the overnight blood culture was taken with a cotton swab and rolled twice over the surface of the agar. Microtubes were completely closed and incubated for 24 h at 37°C in a microaerophilic atmosphere. After incubation, glass tubes and microtubes were sealed with parafilm, while plates were placed inside hermetically sealed plastic bags. Finally, all subcultures were maintained at room temperature until further evaluation.

### 2.4. Evaluation of the Subcultures Viability in Supplemented Agar Types

The detailed conditions of each test are shown in Table 2. In a pilot study, we subcultured* S. pneumoniae *ATCC*49619 *strain onto TH-HYC (*N* = 10) and TH-SYC plates (*N* = 10). The viability of all subcultures was recorded monthly for 6 months.

A second test was performed to evaluate the viability of a group from different sources of pneumococcal strains including 2 clinical isolates and 2 nasopharyngeal isolates, and* S. pneumoniae *ATCC*49619* was included as a control strain. Pneumococci were streaked out onto TH-HYC and TH-SYC plates and five subcultures per strain were set. The viability of all subcultures was recorded monthly for 4 months.

Following the results from the pilot study and the second test, the TH-HYC agar was selected to prove the viability of several pneumococcal serotypes and MLST types in two additional tests. A group of 15 nasopharyngeal pneumococci and* S. pneumoniae *ATCC*49619* strains were inoculated in triplicate onto TH-HYC agar and stored for 2 months into microtubes and for 6 months into glass tubes (test 3 and 4, resp.). The viability of all pneumococcal subcultures was recorded at the end of the tests.

In all tests, the procedure to evaluate pneumococcal subcultures viability was as follows: the amount of visible bacterial growth which could be harvested on one occasion with a 0.001 mL calibrated loop was taken and streaked onto BHI blood agar plate. Subcultures were recorded as viable (at least one visible colony) or nonviable according to the description of Wasas et al., 1998 [13].

### 2.5. Evaluation of the Number of Viable Colony Forming Units (CFU) into Subcultures Stored in Supplemented Agar Types

The number of viable CFU into subcultures stored in both TH-HYC and TH-SYC agar was quantified weekly in one subculture per strain during the pilot study and the second test. The procedure was as follows: the amount of visible growth which could be harvested on one occasion with a 0.001 mL calibrated loop was taken and streaked out onto the surface of BHI blood agar plate. A 4-quadrant pattern was adopted to streak the plates. Plates were incubated for 24 h at 37°C in a microaerophilic atmosphere.

The number of viable CFU was scored on a semiquantitative scale described by O'Brien et al., as follows: scant growth, <25 colonies in quadrant 1; 1+ of growth, ≥25 colonies in quadrant 1 and <25 in quadrant 2; 2+ of growth, ≥25 colonies in quadrant 2 and <25 in quadrant 3; 3+ of growth, ≥25 colonies in quadrant 3 and <25 in quadrant 4; and 4+ of growth, ≥25 colonies in quadrant 4 [14]. Data was further used to perform survival-time curves. Finally, the number of viable CFU was defined as satisfactory (≥2+ or ≥100 CFU) or unsatisfactory (≤1+ growth or <100 CFU) based on the number of colonies from a plate required for techniques such as serotyping, using the criteria described by Pell et al., in 2013 [15].

### 2.6. Evaluation of Pneumococcal Characteristics after Storage in Supplemented Agar Types

At the beginning and at the end of test 2 (after 4 months of storage), we evaluated several phenotypic characteristics in all subcultures. The characteristics evaluated were susceptibility to optochin, bile solubility and the antimicrobial susceptibility according to recommendations [5, 12], and the reaction with Pneumo Latex Agglutination Test DrySpot® (Oxoid) following the manufacturer's instructions. Additionally, we determined pneumococcal serotypes by the capsular reaction test (Quellung reaction) using specific antisera (Statens Serum Institut, Copenhagen, Denmark) [5].

### 2.7. Statistical Analyses

In the pilot study, differences in the number of* S. pneumoniae *ATCC*49619* viable CFU between the two agar types (TH-HYC and TH-SYC) were tested using the unpaired Student *t*-test. In test 2, differences in the means of viable CFU over time between the two agar types (TH-HYC and TH-SYC) and between groups of strains with different sources were tested using a two-way analysis of variance (ANOVA) with Bonferroni posttest correction. Differences between viable CFU of pneumococci with diverse characteristics stored in both agar types were tested using the unpaired Student *t*-test.

Regarding the differences in the contamination rates between media poured into plates compared with agar poured into microtubes and poured into glass tubes, statistical analyses were performed by using two-sided Fisher's exact test. Data were analyzed and graphed with GraphPad Prism v5.0 for Windows (GraphPad Software, San Diego, CA, USA) and Past v3.13 (Natural History Museum, University of Oslo, Oslo, Norway) [16].

## 3. Results

### 3.1. Maintenance of Viability and Characteristics of Pneumococci Stored on Supplemented Agar Types

All pneumococcal subcultures prepared on both TH-HYC and TH-SYC plates remained viable after being stored for 6 months (pilot study) or 4 months (test 2) (Table 3) at room temperature. We observed that pneumococci isolated from patients and carriers (test 2), as well as those displaying diverse serotype and MLST types (tests 3 and 4), were able to survive on supplemented media (Table 3).

Long-term storage did not affect pneumococcal characteristics. Clinical and nasopharyngeal isolates stored during 4 months on both agar types (test 2) and pneumococci with different MLST types, stored during 6 months on TH-HYC agar (test 4), maintained optochin susceptibility. In addition, we could confirm the serotypes of pneumococci stored in both supplemented agar types.

### 3.2. Number of Viable CFU and Survival-Time Curves

**Figure 1:**
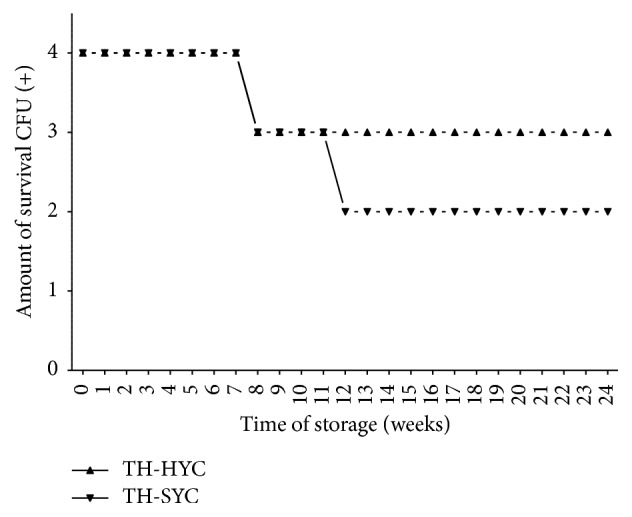
Pneumococcal survival-time curves based on the number of viable CFU of* S. pneumoniae* ATCC 49619 stored on TH-HYC and TH-SYC agar, along the pilot study. CFU (+): the number of CFU was expressed as crosses of growth in the recovery plate. TH-HYC: Todd-Hewitt/Hemoglobin/Yeast/Charcoal. TH-SYC: Todd-Hewitt/Skim-Milk/Yeast/Charcoal.

In the pilot study, the number of* S. pneumoniae *ATCC*49619* viable CFU was satisfactory (2+ or 3+ of growth) for all subcultures after 6 months of storage at room temperature (Figure 1) with no statistical differences between TH-HYC and TH-SYC subcultures (*t, P* = 0.52; 95% CI, 0.105 to 0.934).

**Figure 2:**
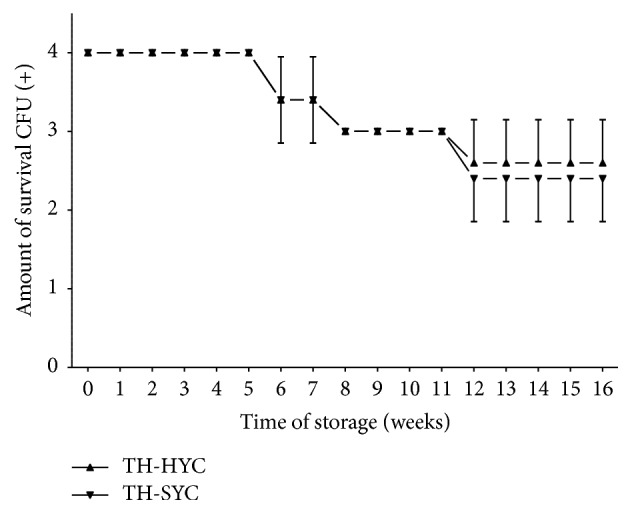
Pneumococcal survival-time curves based on the means and standard deviation of CFU of five pneumococcal strains stored on TH-HYC and TH-SYC agar, along test 2. CFU (+): the number of CFU was expressed as crosses of growth in the recovery plate. TH-HYC: Todd-Hewitt/Hemoglobin/Yeast/Charcoal. TH-SYC: Todd-Hewitt/Skim-Milk/Yeast/Charcoal.

In test 2, both supplemented agar types allowed all pneumococcal strains to maintain a satisfactory number of viable CFU (2+ or 3+ of growth) after 4 months of storage at room temperature (Figure 2) with no statistical differences between means on TH-HYC and means on TH-SYC subcultures (two-way ANOVA; *P* = 0.2772 and *P* = 0.9998).

**Figure 3:**
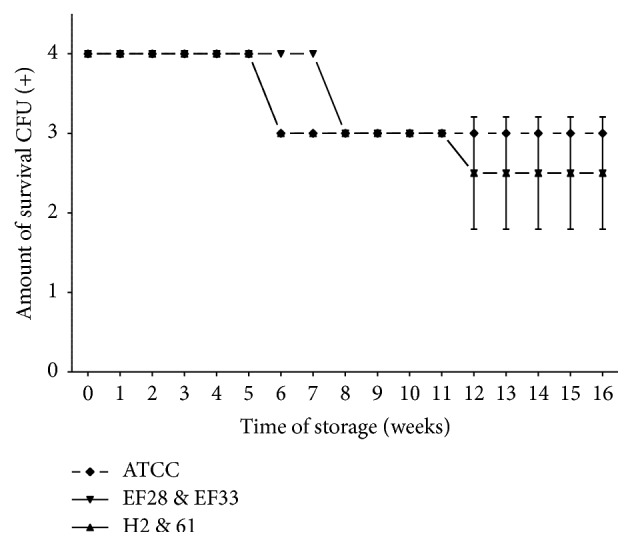
Pneumococcal survival-time curves based on the means and standard deviation of CFU of clinical isolates group (H2 and 61), nasopharyngeal isolates group (EF28 and EF33), and* S. pneumoniae* ATCC 49619 strain stored on TH-HYC agar, along test 2. CFU (+): the number of CFU was expressed as crosses of growth in the recovery plate. TH-HYC: Todd-Hewitt/Hemoglobin/Yeast/Charcoal.

**Figure 4:**
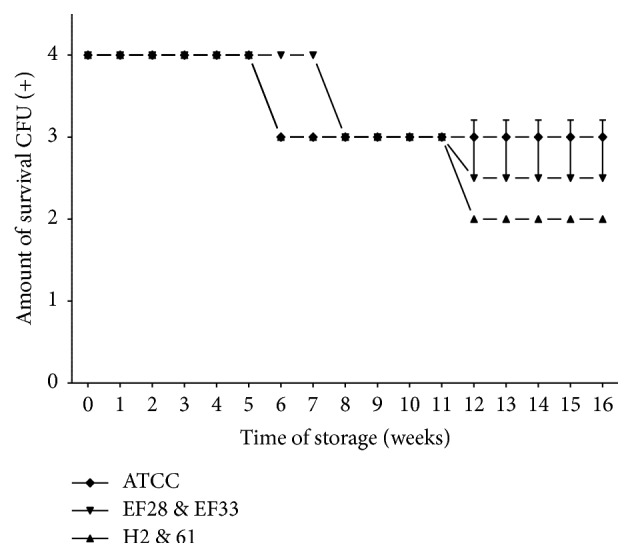
Pneumococcal survival-time curves based on the means and standard deviation of CFU of clinical isolates group (H2 and 61), nasopharyngeal isolates group (EF28 and EF33), and* S. pneumoniae* ATCC 49619 strain stored on TH-SYC agar, along test 2. CFU (+): the number of CFU was expressed as crosses of growth in the recovery plate. TH-SYC: Todd-Hewitt/Skim-Milk/Yeast/Charcoal.

**Figure 5:**
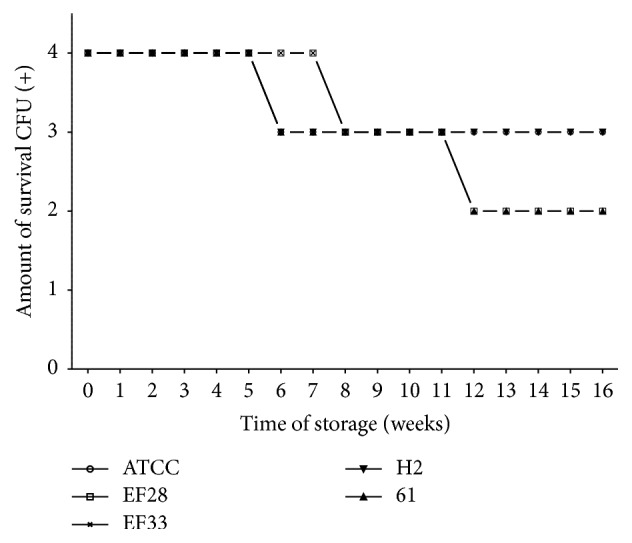
Pneumococcal survival-time curves based on the number of viable CFU of pneumococcal strains: EF28 (serotype 23F), EF33 (nontypeable), H2 (serotype 11), 61 (nontypeable), and* S. pneumoniae* ATCC 49619 strains stored on TH-HYC agar, along test 2 (the number of viable CFU at 16 wk was 2+ for EF28 and 61 strains and 3+ for ATCC, EF33, and H2 strains). CFU (+): the number of CFU was expressed as crosses of growth in the recovery plate. TH-HYC: Todd-Hewitt/Hemoglobin/Yeast/Charcoal.

**Figure 6:**
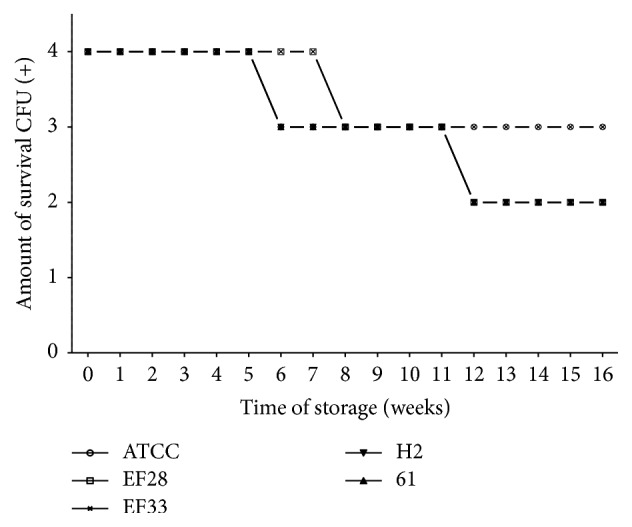
Pneumococcal survival-time curves based on the number of viable CFU of pneumococcal strains: EF28 (serotype 23F), EF33 (nontypeable), H2 (serotype 11), 61 (nontypeable), and* S. pneumoniae* ATCC 49619 strains stored on TH-SYC agar, along test 2 (the number of viable CFU at 16 wk was 2+ for EF28, H2, and 61 strains and 3+ for ATCC and EF33 strains). CFU (+): the number of CFU was expressed as crosses of growth in the recovery plate. TH-SYC: Todd-Hewitt/Skim-Milk/Yeast/Charcoal.

Survival-time curves on TH-HYC and TH-SYC agar (Figures 3, 4, 5, and 6) showed that the decrease of viable CFU occurred slowly over time. In fact, the first decrease of viable CFU (4+ to 3+ of growth) occurred after 6 weeks of storage among clinical isolates and after 8 weeks of storage among the rest of the strains. At week 12, a second decrease of viable CFU (3+ to 2+ of growth) was observed for some strains. Even so, after 4 months of storage on both agar types the number of viable CFU was satisfactory for all strains (2+ or 3+ of growth).

Among subcultures stored on TH-HYC agar (Figure 3) we found no statistical differences in the number of viable CFU over the 4 months between different sources of strains (two-way ANOVA; *P* = 0.3256 and *P* = 0.8881). Indeed, no statistical differences were found among clinical isolates compared with nasopharyngeal isolates (*t, P* = 1; 95% CI, −0.495 to 0.495) and among clinical isolates compared with control strain (*t, P* = 0.067; 95% CI, −0.039 to 0.999). Also, no statistical differences were found among nasopharyngeal isolates compared with control strain (*t, P* = 0.067; 95% CI, −0.039 to 0.999).

It is worth mentioning that, for subcultures stored on TH-SYC agar (Figure 4), we found significant differences in the number of viable CFU over time when groups of strains from different sources were compared (two-way ANOVA; *P* = 0.0003 and *P* = 0.0402). In fact, we observed a very significantly lower number of viable CFU among clinical isolates compared with nasopharyngeal isolates (*t, P* = 0.006; 95% CI, 0.159 to 0.860) and among clinical isolates compared with control strain (*t, P *= 3^−16^; 95% CI, 0.947 to 1.032). However, during the storage on TH-SYC agar we found no differences in the number of viable CFU of nasopharyngeal isolates compared with control strain (*t, P* = 0.067; 95% CI, −0.039 to 0.999).

Survival-time curves of pneumococci with diverse characteristics are separately presented in Figures 5 and 6. Survival-time curves reveal that for most of the strains the number of viable CFU was similar on TH-HYC compared with TH-SYC agar (*t, P* values > 0.9999), except for the clinical isolate serotype 11 (H2 strain) which had a significantly higher number of viable CFU when it was stored on TH-HYC rather than on TH-SYC agar (*t, P* = 0.0105, 95% CI, −0.7744 to −0.1165).

### 3.3. Contamination Rates

Contamination in subcultures stored on plates was high on both agar types. Percentages of contamination at the end of the pilot study (6 month) were 70% versus 80%, and at the end of test 2 (4 month) were 40% versus 58%, in TH-HYC and TH-SYC subcultures, respectively. However, percentages of contamination were lower when TH-HYC subcultures were stored for 2 months into microtubes (*N* = 3/48, 6.25%) or stored for 6 months into glass tubes (*N* = 7/48, 14.58%). Remarkably, the elevated contamination rate observed among subcultures stored onto plates was significantly reduced when subcultures were prepared into glass tubes (pilot study, 70% [*N* = 7/10], versus test 4, 14.58% [*N* = 7/48]; Fisher's exact test, OR = 13.667 [95% CI, 2.836 to 65.860], *P* = 0.0009).

## 4. Discussion

Preservation of strains is of great importance for quality control, teaching, and research. In limited resources countries, where lyophilization and cryopreservation at −70°C are not available, preservation at room temperature is a valid alternative. Wasas et al. described the survival of* S. pneumoniae* subcultures for 6 weeks in Dorset Egg medium [13]. The same study showed that half of the strains stored on CABS medium (Columbia agar base supplemented with 5% horse blood and 0.4% activated charcoal) were no longer viable after 30 days. On the other hand, the two supplemented media tested in this study, agar types TH-HYC and TH-SYC, allow the preservation at room temperature of all pneumococci for, at least, 6 months.

In pneumococcal cultures the products of the metabolism and the activation of the autolysin could cause a decrease of the amount of cells and the loss of viability [17]. Hence, with the purpose of testing supplemented media to preserve viability for long periods, we considered several factors. First, Todd-Hewitt Broth enriched with yeast extract has been often used for pneumococcal growing and it has been demonstrated that it improves the production of capsules [7, 17, 18]. Second, activated charcoal has been widely used as a compound of several transport and culture media as a detoxifying agent and a scavenger of radicals and peroxides [19]. Thus, the addition of activated charcoal to the preservation media could have delayed the beginning of the autolysis process allowing a longer survival rate of pneumococcal cells. Third, contamination is very common on subcultures maintained for long periods [13, 19]. Wasas et al. reported contamination rates ranging from 2% to 23% among pneumococci stored for 6 weeks into screw-cap tubes containing Egg or CABS media [13]. In our work, subcultures on TH-HYC agar showed reasonably low contamination rates of 6.25% and 14.58% after 2 months and 6 months of storage, respectively.

Another important issue is that no matter what method is used, pneumococcal viability decreases greatly over time, even using cryopreservation or lyophilization [4, 20]. Siberry et al., in 2001, found that strains preserved in glycerol-chocolate broth, skimmed milk, rabbit blood, and sheep blood at 4°C became nonviable by the fourth month [4]. In our case, after 4 months of storage, TH-HYC and TH-SYC agar types were able to maintain a satisfactory number of CFU and pneumococci recovered from storage produced the required growth to perform phenotypic techniques such as serotyping (≥2+ of growth or ≥100 CFU) [15].

Interestingly, in test 2 some observations caught our attention. First, on both agar types we observed a significantly faster decrease in the number of viable CFU among clinical isolates compared to nasopharyngeal isolates and control strain. These changes occurred slowly, allowing a satisfactory number of viable CFU (≥2+ of growth) in all groups of strains at least for 4 months. Second, the serotype 11 strain had a significantly higher number of viable CFU on TH-HYC than on TH-SYC agar. At this point, we are unable to say whether the behavior of those pneumococci is related to the source of the strains, to the serotype, or to the individual characteristics of the pneumococci tested. But it seems clear that some biological differences may be evident only under suitable circumstances. Previous studies have found that when pneumococci are maintained for several hours on nutritionally limited broths, serotypes with high colonization prevalence grow sooner than invasive serotypes. Conversely, in nutritionally enriched broths, differences in growth among serotypes could not be seen [7, 9].

We believe that a pertinent aspect to be considered in growing pneumococci is the link between some epidemiological characteristics, such as carriage prevalence and invasive disease potential, with bacterial growth [7, 9]. We should also consider that some serotypes include a variety of clonal lineages, and some clones comprise intraclonal variants with different genetic contents and biological potentials [21, 22]. Following the results from the second test, we strongly aimed at the viability of pneumococci with diverse phenotypic and genetic characteristics. Thus, by carrying out tests 3 and 4 we focus on the media with a better performance (TH-HYC agar). Strains used in these tests comprise serotypes associated with different potential of colonization and invasiveness [1, 8]. For instance, serotypes 14 and 19A have been associated with increased invasive potential, while some serotypes are more associated with colonization as 6B, 19F, 23A, 23F, and nontypeable pneumococci [1, 8]. Beside serotyping, we also put emphasis on testing the viability in pneumococcal strains showing a variety of genetic profiles. First, we tested strains showing the same pneumococcal serotype but related to different international clones, here illustrated by the 6B and 23F serotype strains. Second, we proved the viability among strains related to the same clone but displaying different serotypes, as is the case of Taiwan^19F^-14 clone including 19A and 19F serotype strains. Finally, we tested pneumococci related to England^14^-9, Spain^23F^-1, and Taiwan^23F^-15 clones, showing intraclonal genetic variations revealed by the presence of different transposons. After 6 months of storage, the TH-HYC agar satisfactorily allowed the viability of pneumococci with several serotypes, MLST types, and also having different genetic profiles. Although only a limited number of strains were included in this study, we believe that these are broadly representative of those that circulate in many parts of the world.

Finally, in the literature reviewed we found some concerns related to the loss of some phenotypic characteristics when strains are stored for an extended period. The study by Robson et al. showed that 24% of* S. pneumoniae* isolates turned out to be optochin resistant after long-term storage (>2 years) in 15% glycerol at −70°C. Several authors suggest that, for most isolates, this type of storage is sufficient to induce the optochin-resistant phenotype [23]. Hence, we strongly aimed at the preservation of phenotypic characteristics of the pneumococci used and indeed in this study long-time storage did not change optochin resistance or any other phenotypic pneumococcal characteristic evaluated.

In conclusion, the supplemented media presented here, particularly the TH-HYC agar, are useful for preserving the viability of a variety of pneumococci for at least 6 months at room temperature. Thus, these agar types may be useful for transporting strains between laboratories and are a valid alternative to preserve strains over an extended period of time, especially when methods as cryopreservation or lyophilization are not available.

## Figures and Tables

**Table 1:** Phenotypic and genetic characteristics of *S. pneumoniae* strains.

Strains	Code	Serotype	MLST type^a^	Related international clone^b^	Source	Resistance pattern	Transposons
*First group* *N* = 4	H2	11	ND	ND	CSF^c^	P^f^, T/S	ND
	61	NT	ND	ND	Conjunctivitis^d^	T/S	ND
	EF 28	23 F	ND	ND	Nasopharynx^e^	P^f^	ND
	EF 33	NT	ND	ND	Nasopharynx^e^	E, T, T/S	ND

*Second group* *N* = 15	I-121	6B	90	Spain^6B^-2	Nasopharynx^e^	P^g^, CRO, CXM, MEM, C, CD, E, T, T/S	Tn*5253 *plus Tn*917*
	I-15	6B	90	Spain^6B^-2	Nasopharynx^e^	P^g^, CXM, MEM, CD, E, T, T/S	Tn*5253 *plus Tn*917*
	H-183	6B	135	No	Nasopharynx^e^	P^f^, CD, E, T, T/S	Tn*3872*
	H-222	6B	135	No	Nasopharynx^e^	P^f^, C, T/S	None
	I-206	14	15	SLV England^14^-9	Nasopharynx^e^	CD, E, T/S	Tn*3872*
	H-12	14	2563	DLV England^14^-9	Nasopharynx^e^	CD, E, T	Tn*6002*
	I-196	23F	5165	SLV Spain^23F^-1	Nasopharynx^e^	P^g^, CXM, MEM, C, CD, E, T, T/S	Tn*3872*
	I-246	23F	81	Spain^23F^-1	Nasopharynx^e^	P^g^, CRO, CTX, CXM, FEP, MEM, C, E, T, T/S	Tn*2009*
	I-176	23F	242	Taiwan^23F^-15	Nasopharynx^e^	CD, E, T, T/S	Tn*3872*
	H-59	23F	242	Taiwan^23F^-15	Nasopharynx^e^	E, T, T/S	Tn*2009*
	I-120	23 A	42	DLV Tennessee^23F^-4	Nasopharynx^e^	CD, E, T, T/S	Tn*6002*
	I-248	19F	5167	SLV Taiwan^19F^-14	Nasopharynx^e^	P^g^, CXM, ETP, MEM, CD, E, T, T/S	Tn*2010*
	H-123	19 A	320	DLV Taiwan^19F^-14	Nasopharynx^e^	P^g^, CTX, CXM, ETP, MEM, CD, E, T, T/S	Tn*2010*
	I-29	NT	5168	SLV1106 NCC1	Nasopharynx^e^	P^h^, E, T, T/S	Tn*2009*
	I-34	NT	4837	DLV1106 NCC1	Nasopharynx^e^	P^f^, E, T, T/S	Tn*2009*

^a^MLST: multilocus sequence typing, ^b^NCC: null capsule clade, and PMEN: pneumococcal molecular epidemiology network clone; ^c^strain isolated from cerebrospinal fluid of a child with meningitis with PNC MIC 0.25 *µ*g/mL, ^d^strain isolated from a child with purulent conjunctivitis, ^e^strains isolated from nasopharyngeal carriers aged 0–5 years, ^f^penicillin MIC level of 0.25 *µ*g/mL, ^g^penicillin MIC level of 2 *µ*g/mL, and ^h^penicillin MIC level of 0.50 *µ*g/mL. NT: nontypeable strains, ND: not done, SLV: single-locus variant, DLV: double-locus variant, P: penicillin, CRO: ceftriaxone, CTX: cefotaxime, CXM: cefuroxime, FEP: cefepime, ETP: ertapenem, MEM: meropenem, C: chloramphenicol, CD: clindamycin, E: erythromycin, T: tetracycline, and T/S: trimethoprim-sulfamethoxazole.

**Table 2:** Description of the tests performed.

Test	Strains	Agar	Evaluations	Subcultures	Recipient
*Test 1 (pilot study)* (6 months)	Total = 1 *S. pneumoniae *ATCC *49619*	TH-HYC TH-SYC	(i) Viability of all subcultures (monthly) (ii) Amount of viable CFU in 1 subculture per strain in both media (weekly)	*N* = 10 per strain Total: 10 per medium	Petri dishes (15 × 100 mm)

*Test 2* Clinical and nasopharyngeal isolates (4 months)	Total = 5 Meningitis isolate *N* = 1 Conjunctivitis isolate *N* = 1 Nasopharyngeal strains *N* = 2 *S. pneumoniae *ATCC *49619*	TH-HYC TH-SYC	(i) Viability of all subcultures (monthly) (ii) Amount of viable CFU in 1 subculture per strain in both media (weekly) (iii) Maintenance of serotypes, optochin susceptibility, reaction with agglutination test, and patterns of resistance in all subcultures (at the end of the test)	*N* = 10 per strain Total: 50 per medium	Petri dishes (15 × 100 mm)

*Test 3* several serotypes and MLST types (2 months)	Total = 16 Nasopharyngeal strains *N* = 15 *S. pneumoniae *ATCC *49619*	TH-HYC	(i) Viability of all subcultures (at the end of the test) (ii) Maintenance of optochin susceptibility, reaction with agglutination test, patterns of resistance, and presence of macrolide resistant genes in all subcultures (at the end of the test)	*N* = 3 per strain Total: 48	Safe-lock microtubes (1.5 mL)

*Test 4* several serotypes and MLST types (6 months)	Total = 16 Nasopharyngeal strains *N* = 15 *S. pneumoniae *ATCC *49619*	TH-HYC	(i) Viability of all subcultures (at the end of the test) (ii) Maintenance of optochin susceptibility, reaction with agglutination test, patterns of resistance, and presence of macrolide resistant genes in all subcultures (at the end of the test)	*N* = 3 per strain Total: 48	Screw-cap glass tubes (20 × 150 mm)

TH-HYC: Todd-Hewitt/Hemoglobin/Yeast/Charcoal. TH-SYC: Todd-Hewitt/Skim-Milk/Yeast/Charcoal. MLST: multilocus sequence typing.

**Table 3:** Viability and contamination of pneumococcal subcultures stored on two supplemented media.

Test	Month	Subcultures on TH-HYC agar			Subcultures on TH-SYC agar		
		Contaminated *N*	Evaluated for viability^a^ *N*	Viable^b^ %	Contaminated *N*	Evaluated for viability^a^ *N*	Viable^b^ %
*Test 1 (pilot study)* (*N* = 10 subcultures per medium into plates)	1°	0	10	100	0	10	100
	2°	0	10	100	0	10	100
	3°	2	8	100	3	7	100
	4°	3	7	100	3	7	100
	5°	5	5	100	6	4	100
	6°	7	3	100	8	2	100

*Test 2* (*N* = 50 subcultures per medium into plates)	1°	0	50	100	5	45	100
	2°	9	41	100	14	36	100
	3°	11	39	100	3	17	100
	4°	20	30	100	29	21	100

*Test 3* (*N* = 48 subcultures into microtubes)	2°	3	45	100	NA	NA	NA

*Test 4* (*N* = 48 subcultures into glass tubes)	6°	7	41	100	NA	NA	NA

Subcultures were recorded as viable (at least one visible colony) or nonviable according to the description of Wasas et al., in 1998. ^a^Subcultures with no contamination at the moment of the evaluation. ^b^Percentages based on the number of cultures with no contamination at the moment of the evaluation. TH-HYC: Todd-Hewitt/Hemoglobin/Yeast/Charcoal. TH-SYC: Todd-Hewitt/Skim-Milk/Yeast/Charcoal. NA: not applicable.
